# Discussion on protein recommendations for supporting muscle and bone health in older adults: a mini review

**DOI:** 10.3389/fnut.2024.1394916

**Published:** 2024-05-22

**Authors:** Inge Groenendijk, Lisette C. P. G. M. de Groot, Inge Tetens, Pol Grootswagers

**Affiliations:** ^1^Division of Human Nutrition and Health, Wageningen University & Research, Wageningen, Netherlands; ^2^Department of Nutrition, Exercise and Sports, University of Copenhagen, Copenhagen, Denmark

**Keywords:** protein, amino acid, aging, muscle, bone, physical function

## Abstract

Muscle and bone tissues are interconnected, and both rely on an adequate protein intake. Recommendations for protein intake for older adults specifically vary across countries. The purpose of this narrative review is to discuss the existing evidence for protein recommendations for supporting muscle and bone health in older adults and to evaluate if a protein intake above the current population reference intake (PRI) for older adults would be scientifically justified. First, this review summarizes the protein recommendations from bodies setting dietary reference values, expert groups, and national health organizations. Next, relevant studies investigating the impact of protein on muscle and bone health in older adults are discussed. In addition, the importance of protein quality for muscle and bone health is addressed. Lastly, a number of research gaps are identified to further explore the added value of a protein intake above the PRI for older adults.

## Introduction

Declining muscle mass and strength and bone mineral density (BMD) are common during aging (1). These changes may lead to the development of sarcopenia and osteoporosis. Muscle and bone are interconnected to each other. From a mechanistic point of view, the mechanical forces exerted by muscles during certain activities play a crucial role in stimulating bone formation (2). In addition, osteoporosis and sarcopenia share risk factors and often occur within the same individual (3). Additionally, low muscle mass and strength that occur with sarcopenia increase the risk of falls, and subsequently the risk of fractures, and fractures can in turn accelerate muscle mass loss (4). Protein plays a vital role in supporting both muscle and bone health in older adults; an adequate protein quantity and quality has been proposed to maintain muscle and bone health later in life (5–7).

Recommendations for protein intake for older adults vary across countries (8–12). Some bodies setting dietary reference values and national health organizations make no distinction between adults and older adults (11–14), while others advocate for higher protein intake recommendations specifically tailored to the needs of older adults (8–10). This narrative review first summarizes the current national and international protein recommendations for (older) adults. Next, we describe recent and relevant studies focusing on the impact of protein on muscle and bone health in older adults specifically. In addition, the importance of protein quality is addressed. Thus, the purpose of this narrative review is to discuss the existing evidence for protein recommendations for supporting muscle and bone health in older adults and to evaluate if a protein intake above the population reference intake (PRI) of 0.83 g/kg/d for older adults would be scientifically justified.

## Current protein recommendations

The current protein recommendations from EFSA (13) and the FAO/WHO/UNU (14) are based on a meta-analysis of nitrogen balance studies from 2003, involving 19 studies and 235 healthy adults (15). Hereby the estimated average requirement (EAR) was established at 0.65 g/kg bodyweight/d (g/kg/d) and the PRI at 0.83 g/kg/d. In a stratified analysis comparing younger adults (<40 years, *n* = 221) with older adults (>67 years, *n* = 14), a statistically non-significant difference in the EAR of 27 mg N/kg/d (=0.17 g protein/kg/d) was found (15). Since only 14 older adults were included, the power of this comparison is inadequate. The meta-analysis was repeated in 2014 and led to the same conclusions (16). However, still only 54 older adults (≥60 years) could be included. In both meta-analyses the sample size of older adults was low, so there was limited power to establish if there was a difference between young and older adults. Thus, more nitrogen balance studies involving older adults are needed. Instead of using nitrogen balance studies in which the requirement for protein is based on the lowest amount of dietary protein intake that will balance the nitrogen losses from the body (14), focusing on the effect of protein on physiological variables, such as muscle mass, physical functioning and bone health, in older adults may be of additional value for the estimation of protein recommendations.

The nitrogen balance-based recommendations have been adopted by several national and international organizations (13, 14, 17). However, several expert groups including the PROT-AGE Study Group (5) and European Society for Clinical Nutrition and Metabolism (ESPEN) (6) advise higher protein intakes based on evidence for maintaining muscle mass and function. The PROT-AGE study Group (5) recommends for older adults an average daily intake of at least 1.0 to 1.2 g/kg/d to maintain and regain lean body mass and function. In case of an acute or chronic disease, even higher intakes are proposed (1.2–1.5 g/kg/d). This is in line with the recommendations of the ESPEN expert group (6): at least 1.0–1.2 g/kg/day for healthy older people, and 1.2–1.5 g/kg/day for older people who are malnourished or at risk of malnutrition because they have acute or chronic illness.

Some national organizations also revised their protein recommendations for older adults. For example, the Nordic countries changed the recommended protein intake into 1.2 g/kg/day in 2012 (8), which was maintained in the 2023 version (18), and the nutrition societies of Germany, Austria, and Switzerland revised it to 1.0 g/kg/d for adults >65 years in 2019 (9). In 2021, the Food Safety Authority of Ireland advises older adults at risk of frailty, sarcopenia, or undernutrition to consume a minimum of 1.0–1.2 g/kg/d (10). These increases in national recommendations are based on an overall assessment of metabolic and functional parameters. However, due to different criteria, for example the type of studies used (nitrogen balance studies, cohorts, RCTs), organizations in the Netherlands and United Kingdom conclude that the evidence is still insufficient to increase the recommendation ≥0.8 g/kg/d (11, 12).

Regarding bone health, European guidance provided by International Osteoporosis Foundation (IOF) and European Society for Clinical and Economic Aspects of Osteoporosis, Osteoarthritis and Musculoskeletal Diseases (ESCEO) stated in 2013 that 1.0 g/kg/d of protein can be recommended in the general management of patients with osteoporosis (19, 20). However, in the updated guidance of 2019 this number is removed and changed to “sufficient dietary protein” (21). In 2023, the first set of dietary recommendations in the prevention and treatment of osteoporosis have been published by the French Rheumatology Society and the Osteoporosis Research and Information Group (22). Based on evidence from cohort studies, a protein intake of at least 1.0–1.2 g/kg/day (with “high quality” animal proteins) as part of a balanced diet with adequate calories, calcium and vitamin D intakes is advised (19, 22).

## Health effects of a protein intake above the PRI in older adults

The most recent systematic review on the health effects of increasing protein intake above the current PRI in older adults was published by the Health Council of the Netherlands in 2022 (11). Data of >1,300 subjects (≥60 years or mean ≥ 65 years) from 18 RCTs were included and only RCT’s lasting ≥4 weeks were included. The Health Council concluded that an increased protein intake, combined with physical exercise, has a possible beneficial effect on lean body mass, while an increased protein intake without exercise had *likely no effect* on muscle strength, physical function, and bone health. However, three limitations are of note (23). First, only studies with (relatively) healthy older adults could be included. Studies with older adults living in a nursing home or care home were not eligible and studies in which the study population consisted of hospitalized or immobilized patients or of individuals with a specific disease were excluded. However, the population of older adults is very heterogeneous. Considering the prevalence of malnutrition (9%) (24), frailty (11%) (25), sarcopenia (10–27%) (26), obesity (35%) (27) and multimorbidity (51%) (28), the results of this systematic review only apply to the healthy segment of the older population. Second, no cohort studies were included. In research on bone health, cohort studies significantly contribute to our understanding of factors associated with bone health, since intervention studies are typically too short of duration to identify changes in BMD. Therefore, high-quality cohort studies should have a prominent role in evaluating the role of a protein intake above the PRI on bone health in older adults and should be included in the estimation of reference values for protein. Third, studies with a non-isocaloric control intervention were excluded. This strict criterion excluded many placebo-controlled RCTs, while the protein supplements increase energy intake only by about 80–160 kcal per day. Such a minimal amount of extra calories is not expected to affect fat free mass or physical functioning to an extent warranting exclusion of these important trials.

### Muscle health

We have identified five relevant RCTs investigating the impact of enhancing protein intake on muscle health that are excluded from the systematic review of the Health Council of the Netherlands or published after their systematic search. These RCTs used a non-isocaloric control group but do have added value to the scientific evidence. These include the studies of *ProMuscle* (29, 30), *ProMuscle in Practice* (31, 32), *ProMuscle Implementation* (33), *PROMISS* (34, 35), and *CALM* (36). All investigated the effect of protein on muscle health outcomes, and found beneficial effects compared to control groups (Table 1). In *ProMuscle* (29, 30), there were four arms: supplementation with 31 g of milk protein concentrate or placebo, with or without resistance exercise training for 24 weeks. It was found that lean body mass increased in the protein+exercise group. Strength and physical performance increased in the protein only group and exercise groups. In *ProMuscle in Practice* (31, 32), an increased protein intake of 25 g per main meal in combination with resistance exercise training was investigated for 24 weeks. Lean body mass, muscle strength and the Short Physical Performance Battery score were improved in the protein and exercise group compared to the control group. *ProMuscle Implementation* (33) showed that tailored nutrition advice aiming at 20-25 g protein per main meal for 12 weeks combined with resistance exercise training improved chair-rise performance and leg strength. In *PROMISS* (34, 35), 400-m walking time and leg extension strength improved in the intervention groups (dietary advice to increase protein intake to ≥1.2 g/kg aBW/d with or without advice to time protein intake in close proximity of usual physical activity) compared to controls (no dietary advice). In the *CALM* study (36), the effect of protein supplementation (20 g, twice daily), with or without resistance exercise training for 1 year, was investigated and compared with a placebo (carbohydrate). Quadriceps size and leg strength improved in the exercise+protein group compared to the protein only and placebo group. In the protein only group, there were no improvements in muscle health over time compared to placebo. Despite that the baseline protein intake in these five studies was already >0.8 g/kg/d, beneficial effects on muscle health outcomes were found, especially when combined with exercise training. Even larger effects may be expected in older adults with lower protein intakes (<0.8 g/kg/d).

**Table 1:** Characteristics and results of five studies investigating the effect of protein on muscle health.

Study [ref]	Population	Intervention	Baseline protein intake (g/kg/d)	Results^1^
ProMuscle (*n* = 127) (29, 30)	≥65 years; frail or pre-frail according to fried criteria	Four arms: supplementation with 31 g of milk protein concentrate or placebo, with or without RET for 24 weeks	Mean (95% CI) Protein: 1.0 (0.9–1.1) Placebo: 1.0 (0.9–1.1) Mean ± SEM Protein+exercise: 1.0 ± 0.0 Placebo+exercise: 1.0 ± 0.0	Protein compared to placebo: physical performance increased, *p* = 0.02^2^; LBM stable, *p* > 0.05^2^; strength improved in both groups, p < 0.01^3^, but no interaction. Protein+exercise compared to placebo+exercise: LBM increased, *p* = 0.006^2^; strength and physical performance improved in both groups, *p* < 0.001^3^, but no interaction.
ProMuscle in practice (*n* = 168) (32)	≥65 years; frail or pre-frail according to Fried criteria or physical inactive and experiencing difficulties in daily activities	Two arms: increased protein intake of 25 g per main meal via conventional and enriched products combined with RET versus control for 24 weeks	Mean (95% CI) Control: 1.08 (1.01–1.15) Intervention: 1.12 (1.05–1.19)	LBM: 0.4 (0.1–0.8) kg, *p* < 0.05^2^ Muscle strength: 30.8 (11.5–50.0) N, *p* < 0.01^2^ SPPB score: 0.5 (0.1–0.9), *p* < 0.05^2^
ProMuscle implementation (*n* = 35) (33)	≥65 years; room for improving muscle strength or protein intake, or recovery after inactive period	One arm: tailored nutrition advice aiming at 20–25 g protein per main meal for 12 weeks combined with RET	Not measured	Chair-rise performance: −3.3 ± 4.2 s, *p* = 0.001^3^ Leg strength: 47.8 ± 46.8 kg, *p* < 0.001^3^
PROMISS (*n* = 276) (35)	≥65 years; habitual protein intake < 1.0 g/kg aBW/d at baseline	Three arms: dietary advice to increase protein intake to ≥1.2 g/kg aBW/d with (PROT+TIMING) or without (PROT) advice to time protein intake in close proximity of usual physical activity, or no dietary advice for 24 weeks	Mean ± SE PROT+TIMING: 0.81 ± 0.01 PROT: 0.82 ± 0.01 Control: 0.82 ± 0.01	Compared to controls: 400-m walking time PROT+TIMING: −4.9 (−14.5 to 4.7) s PROT: −12.4 (−21.8 to −2.9) s Leg extension strength PROT+TIMING: 24.3 (0.2–48.5) N PROT: 32.6 (10.6–54.5) N
CALM (*n* = 76) (36)	>65 years; no medical condition potentially preventing them from safely completing the intervention	Three arms: Supplementation with 20 g of whey protein 2 times/day with or without heavy RET, or placebo (maltodextrin+sucrose) for 12 months	Mean ± SD Protein: 1.1 ± 0.3 Protein+exercise: 1.1 ± 0.4 Placebo: 1.2 ± 0.3	Protein only compared to placebo: no improvements in quadriceps size, lower extremity strength and power, functional capabilities, and body composition. Compared to protein only, protein+exercise improved in: Quadriceps size: +1.68 (0.41–2.95) cm^2^, *p* = 0.03^4^ Dynamic knee extensor strength: +18.4 (10.1–26.6) Nm, *p* < 0.001^4^ Isometric knee extensor strength: +23.9 (14.2–33.6) Nm, *p* < 0.001^4^

LBM, lean body mass; RET, resistance exercise training; SPPB, Short Physical Performance Battery.

^1^Values are mean ± SD or *β* (95% CI), unless stated otherwise.

^2^*p* value for time × treatment effect.

^3^*p* value from paired samples *t*-tests.

^4^Values are mean between-group difference (95% CI).

### Bone health

Regarding bone health, the latest systematic review and meta-analysis on dietary protein intake and bone health in older adults was published in 2019 by Groenendijk et al. (7). The systematic review showed a positive trend between higher protein intakes (above the PRI) and higher femoral neck and total hip BMD. The meta-analysis showed that a higher protein intake resulted in a significant decrease in hip fractures of 11%. Since then, new studies investigating the relationship between protein and bone health in older adults have been published (repeated search 02/01/2024) (19). At least one observational study by Weaver et al. (37) and one intervention study by Kemmler et al. (38) would have been included if the systematic review would be repeated (Table 2). Weaver et al. (37) showed that older adults with higher protein intake (mean ± SD: 1.1 ± 0.4 g/kg/d) had 1.8% higher mean hip and 6.0% higher lumbar spine BMD at baseline compared to those with a lower protein intake (0.8 ± 0.3 g/ kg/d). While the higher and lower protein intake groups had similar BMD changes over 4 years of follow-up, the higher protein intake group had a 64% (95% CI: 0.14, 0.97) reduced risk of vertebral fractures during 5 years of follow-up. Kemmler et al. investigated the effects of 18-months of high intensity dynamic resistance exercise and whey protein supplementation on BMD in older men with osteoporosis and sarcopenia (38). Total protein intake was aimed at 1.5–1.6 g/kg/d in the intervention group and 1.2 g/kg/d in the control group. While the intervention group followed a supervised high intensity training program twice a week, the control group received no exercise program. In the intervention group, BMD at the lumbar spine and total hip was higher than the control group after 18 months (mean difference 0.012 and 0.013 mg/cm^2^, respectively). Both studies support the hypothesis that a protein intake above the PRI improves bone health in older adults (19). Note that only healthy older adults were included in the systematic review by Groenendijk et al. and the two recent studies. Different results may be seen in for example frail, undernourished, or osteoporotic individuals.

**Table 2:** Characteristics and results of two recent studies investigating the effect of protein on bone health in healthy older adults.

Study (ref)	Population	Follow-up period (cohort) or intervention (trial)	Baseline protein intake (g/kg/d)^1^	Results
Weaver et al. (37) (*n* = 3,075)	70–79 years; healthy, white and Black men and women	5 years of follow-up	High: 1.1 ± 0.4 Low: 0.8 ± 0.3	Compared to low protein group, high protein group had: 1.8% higher mean hip BMD at baseline, *p* < 0.05 6.0% higher lumbar spine BMD at baseline, *p* < 0.05 No differences in BMD after 4 years Reduced risk of vertebral fractures after 5 years, HR 0.64 (95% CI: 0.14–0.97), *p* = 0.04
Kemmler et al. (38) (*n* = 43)	≥72 years; men with osteoporosis and sarcopenia	Whey protein supplementation and high intensity dynamic RET for 18 months	Control: 1.29 ± 0.34 Intervention: 1.10 ± 0.25	Compared to control, intervention group improved in: Lumbar spine BMD: +0.012 (0.001 to −0.020) mg/cm^2^, *p*=0.024^2^ Total hip BMD: +0.013 (0.002–0.022) mg/cm^2^, *p*=0.025^2^

HR, hazard ratio; RET, resistance exercise training.

^1^Values are mean ± SD.

^2^Values are mean difference (95%CI). *p* value from dependent *t*-tests.

## Protein quality

As dietary guidelines increasingly advocate for more plant-based diets to address environmental sustainability and health concerns (39–43), a critical view on protein quality is needed, especially for older adults. Bones and muscles require a continuous supply of amino acids for maintenance and repair. The alkaline nature of plant-based diets may offer some bone health advantages by reducing the acid load on the body (by increasing potassium intake), potentially mitigating bone loss (44). However, the lower anabolic potential of plant proteins has been suggested to compromise muscle mass, muscle strength, and bone health (45, 46).

Protein quality, determined by the digestibility and the amino acid pattern of a protein, is generally lower in plant protein sources than in animal protein sources. Such lower quality might lead to reductions in lean body mass, including muscle and bone tissue (45). Some observational studies showed lower BMD values in vegetarians, vegans and older adults with a low animal:plant protein ratio (51:49) (46, 47). In addition, vegetarians and vegans have been shown to have an increased fracture risk, potentially due to intakes below the average requirement of protein, calcium and/or vitamin B12 (47, 48). RCTs with longer term vegan diets in older adults and specific measures of body composition, BMD and bone turnover are urgently needed, and some of these trials have been initiated (ClinicalTrials.gov ID NCT05809466 and NCT06130956).

In response to the lower quality of plant proteins, there’s a prevailing recommendation to increase total protein intake (with a factor of 1.3) to meet physiological needs on a plant-based diet (49). However, this approach may not be feasible for older adults, who often experience diminished appetite and energy intake capacity (45). A more viable strategy involves smarter meal planning that leverages the concept of protein complementation—combining different plant protein sources to achieve a complete amino acid profile. This approach can enhance the overall quality of the protein consumed without necessitating increased food intake. For example, combining legumes (high in lysine, low in methionine) with cereal grains (high in methionine, low in lysine) can provide a more balanced amino acid profile, increasing the quality of the total meal protein. Such dietary strategies, however, require careful planning and knowledge, highlighting the need for tools that help dietitians, meal planners and consumers to find optimal combinations of plant-based proteins, which our group is currently working on (50).

It could be that essential amino acids in protein sources exert direct effects on bone health, similar to the way in which leucine can stimulate muscle protein synthesis (51, 52). In 2022, evidence on the potential role of essential amino acids on bone aging was gathered (53). The authors report that *in vivo* and *in vitro* studies showed that several essential amino acids (lysine, threonine, methionine, tryptophan, and isoleucine) can increase osteoblast proliferation, activation, and differentiation, and decrease osteoclast activity, but that conflicts in mechanisms of action exists (53). These findings were partly replicated in human studies. In an observational study (*n* = 2,997, mean age 72 years), higher serum concentrations of valine, leucine, isoleucine and tryptophan were associated with less hip BMD decline after 4 years (OR/SD ranging from 0.83 to 0.92) after multiple adjustments (54). In that cohort, higher serum tryptophan concentrations were also associated with fewer major osteoporotic fractures (HR/SD 0.86) (54), a finding that has been confirmed in another cohort study (hip fracture cases *n* = 131; controls *n* = 131) (55). Alternatively, in a study using UK Biobank data (*n* = 111,257; 901 hip fracture cases) that investigated the association between circulating amino acids and incident fractures, an association was found between valine concentrations and hip fractures (HR/SD 0.79) (56). This finding was replicated in the *Umeå Fracture and Osteoporosis hip fracture study* (hip fracture cases *n* = 2,225; controls *n* = 2,225) (56). Although the evidence is starting to suggest a protective role of essential amino acid concentrations, specifically valine and tryptophan, more high-quality evidence is required to arrive at firm conclusions about the role of essential amino acids in bone health.

## Discussion

The differences in protein recommendations for older adults between bodies setting dietary reference values, expert groups, and national health organizations stem from the utilization of distinct criteria. When nitrogen balance studies are used, the conclusion is to set the PRI at 0.83 g/kg/d. But if physiological outcomes are taken into account, then it is time to reconsider the values, which is acknowledged by other critical reviews as well (57, 58). Another criterium is if cohort studies are valued to the same extent as RCTs. Especially for bone health, the evidence originates mostly from cohort studies.

Physiological changes that occur with aging, such as sarcopenia, osteoporosis, reduced protein synthesis, and altered metabolism, make it probable that a protein intake above the PRI is needed for older adults compared to adults. To justify this higher protein recommendation for older adults, a number of research gaps needs to be addressed. First, more nitrogen balance studies need to be performed in older adults. Additionally, a distinction between different age groups within the older population should be made, for example between individuals who are between 65 and 80 years and those who are above the age of 80 years. This differentiation allows for a more nuanced understanding of the effects of aging and the potential variations in health needs and outcomes. Secondly, research should focus on the effect of protein on physiological and clinically relevant outcomes. These outcomes are also highly valued by the older population since they directly influence quality of life and overall well-being. Well-designed, large, and long-term RCTs are especially needed to determine if a protein intake above the PRI can support bone health and/or prevent osteoporosis, as evidence from trials is limited. Thirdly, the heterogeneity of the older population needs to be acknowledged. Individuals in this demographic vary widely in terms of health status. For example, different recommendations may be necessary for those who are malnourished or for those who have several comorbidities.

Next, to protein quantity, more studies are needed to investigate protein quality. While plant-based diets offer environmental and health benefits, their adoption among older adults raises concerns regarding protein and nutrient adequacy for muscle and bone health. Unraveling the true effect of plant-based diets on muscle and bone health in older adults is needed, as well as solutions to improve the protein quality of plant-based diets. An exciting topic for future exploration is the initial evidence that hints at a protective role of essential amino acids in bone health.

In conclusion, considering physiological and clinically relevant outcomes in protein recommendations for older adults is preferable, focusing on both the quantity and quality of protein.

## Author contributions

IG: Conceptualization, Investigation, Visualization, Writing – original draft, Writing – review & editing. LG: Conceptualization, Investigation, Supervision, Validation, Writing – review & editing. IT: Validation, Writing – review & editing. PG: Conceptualization, Investigation, Supervision, Writing – original draft, Writing – review & editing.

## References

[1] Jaul E Barron J. Age-related diseases and clinical and public health implications for the 85 years old and over population. Front Public Health. (2017) 5:335. doi: 10.3389/fpubh.2017.00335, PMID: 29312916 PMC5732407

[2] Novotny SA Warren GL Hamrick MW. Aging and the muscle-bone relationship. Physiology. (2015) 30:8–16. doi: 10.1152/physiol.00033.2014, PMID: 25559151 PMC4285576

[3] Curtis E Litwic A Cooper C Dennison E. Determinants of muscle and bone aging. J Cell Physiol. (2015) 230:2618–25. doi: 10.1002/jcp.25001, PMID: 25820482 PMC4530476

[4] Bonjour J-P. The dietary protein, IGF-I, skeletal health axis. Horm Mol Biol Clin Invest. (2016) 28:39–53. doi: 10.1515/hmbci-2016-0003, PMID: 26985688

[5] Bauer J Biolo G Cederholm T Cesari M Cruz-Jentoft AJ Morley JE. Evidence-based recommendations for optimal dietary protein intake in older people: a position paper from the PROT-AGE study group. J Am Med Dir Assoc. (2013) 14:542–59. doi: 10.1016/j.jamda.2013.05.021, PMID: 23867520

[6] Deutz NE Bauer JM Barazzoni R Biolo G Boirie Y Bosy-Westphal A. Protein intake and exercise for optimal muscle function with aging: recommendations from the ESPEN expert group. Clin Nutr. (2014) 33:929–36. doi: 10.1016/j.clnu.2014.04.007, PMID: 24814383 PMC4208946

[7] Groenendijk I den Boeft L van Loon LJC de Groot L. High versus low dietary protein intake and bone health in older adults: a systematic review and Meta-analysis. Comput Struct Biotechnol J. (2019) 17:1101–12. doi: 10.1016/j.csbj.2019.07.005, PMID: 31462966 PMC6704341

[8] Nordic Council of Ministers. Nordic nutrition recommendations 2012: Integrating nutrition and physical activity. 5th ed. Copenhagen: Nordisk Ministerråd (2014).

[9] Richter M Baerlocher K Bauer JM Elmadfa I Heseker H Leschik-Bonnet E. Revised reference values for the intake of protein. Ann Nutr Metab. (2019) 74:242–50. doi: 10.1159/000499374, PMID: 30904906 PMC6492513

[10] Food Safety Authority of Ireland. Scientific recommendations for food-based dietary guidelines for older adults in Ireland. Dublin: Food Safety Authority of Ireland (2021).

[11] Hengeveld LM de Goede J Afman LA Bakker SJL Beulens JWJ Blaak EE. Health effects of increasing protein intake above the current population reference intake in older adults: a systematic review of the health Council of the Netherlands. Adv Nutr. (2022) 13:1083–117. doi: 10.1093/advances/nmab140, PMID: 35016214 PMC9340973

[12] Public Health England. Government dietary recommendations. London: Public Health England (2016).

[13] EFSA Panel on Dietetic Products Nutrition and Allergies. Scientific opinion on dietary reference values for protein. EFSA J. (2012) 10:2557. doi: 10.2903/j.efsa.2012.2557

[14] Joint WHO/FAO/UNU Expert Consultation. Protein and amino acid requirements in human nutrition. Geneva: World Health Organization (2007). 18330140

[15] Rand WM Pellett PL Young VR. Meta-analysis of nitrogen balance studies for estimating protein requirements in healthy adults. Am J Clin Nutr. (2003) 77:109–27. doi: 10.1093/ajcn/77.1.109 12499330

[16] Li M Sun F Piao JH Yang XG. Protein requirements in healthy adults: a meta-analysis of nitrogen balance studies. Biomed Environ Sci. (2014) 27:606–13. doi: 10.3967/bes2014.093, PMID: 25189607

[17] Health Council of the Netherlands. Dietary reference values for proteins. The Hague: Health Council of the Netherlands (2021).

[18] Blomhoff R Andersen R Arnesen EK Christensen JJ Eneroth H Erkkola M. Nordic nutrition recommendations 2023. Copenhagen: Nordic Council of Ministers (2023).

[19] Groenendijk I. Nutritional factors to support bone health in older adults. Wageningen: Wageningen University (2023).

[20] Kanis JA McCloskey EV Johansson H Cooper C Rizzoli R Reginster JY. European guidance for the diagnosis and management of osteoporosis in postmenopausal women. Osteoporos Int. (2013) 24:23–57. doi: 10.1007/s00198-012-2074-y, PMID: 23079689 PMC3587294

[21] Kanis JA Cooper C Rizzoli R Reginster JY. European guidance for the diagnosis and management of osteoporosis in postmenopausal women. Osteoporos Int. (2019) 30:3–44. doi: 10.1007/s00198-018-4704-5, PMID: 30324412 PMC7026233

[22] Biver E Herrou J Larid G Legrand MA Gonnelli S Annweiler C. Dietary recommendations in the prevention and treatment of osteoporosis. Joint Bone Spine. (2023) 90:105521. doi: 10.1016/j.jbspin.2022.105521, PMID: 36566976

[23] van der Meij BS Grootswagers P de Groot LCPGM de van der Schueren MAE. Experts reageren op Voedingsnormen voor eiwitten voor ouderen. Nederlands Tijdschrift voor Voeding en Dietetiek. (2021) 76:39–41.

[24] Leij-Halfwerk S Verwijs MH van Houdt S Borkent JW Guaitoli PR Pelgrim T. Prevalence of protein-energy malnutrition risk in European older adults in community, residential and hospital settings, according to 22 malnutrition screening tools validated for use in adults ≥65 years: a systematic review and meta-analysis. Maturitas. (2019) 126:80–9. doi: 10.1016/j.maturitas.2019.05.006 31239123

[25] Collard RM Boter H Schoevers RA Oude Voshaar RC. Prevalence of frailty in community-dwelling older persons: a systematic review. J Am Geriatr Soc. (2012) 60:1487–92. doi: 10.1111/j.1532-5415.2012.04054.x, PMID: 22881367

[26] Petermann-Rocha F Balntzi V Gray SR Lara J Ho FK Pell JP. Global prevalence of sarcopenia and severe sarcopenia: a systematic review and meta-analysis. J Cachexia Sarcopenia Muscle. (2022) 13:86–99. doi: 10.1002/jcsm.12783, PMID: 34816624 PMC8818604

[27] Samper-Ternent R Al SS. Obesity in older adults: epidemiology and implications for disability and disease. Rev Clin Gerontol. (2012) 22:10–34. doi: 10.1017/s0959259811000190, PMID: 22345902 PMC3278274

[28] Chowdhury SR Chandra Das D Sunna TC Beyene J Hossain A. Global and regional prevalence of multimorbidity in the adult population in community settings: a systematic review and meta-analysis. EClinicalMedicine. (2023) 57:101860. doi: 10.1016/j.eclinm.2023.101860, PMID: 36864977 PMC9971315

[29] Tieland M van de Rest O Dirks ML van der Zwaluw N Mensink M van Loon LJ. Protein supplementation improves physical performance in frail elderly people: a randomized, double-blind, placebo-controlled trial. J Am Med Dir Assoc. (2012) 13:720–6. doi: 10.1016/j.jamda.2012.07.005, PMID: 22889730

[30] Tieland M Dirks ML van der Zwaluw N Verdijk LB van de Rest O de Groot LC. Protein supplementation increases muscle mass gain during prolonged resistance-type exercise training in frail elderly people: a randomized, double-blind, placebo-controlled trial. J Am Med Dir Assoc. (2012) 13:713–9. doi: 10.1016/j.jamda.2012.05.020, PMID: 22770932

[31] van Dongen EJI Haveman-Nies A Wezenbeek NLW Dorhout BG Doets EL de Groot L. Effect, process, and economic evaluation of a combined resistance exercise and diet intervention (ProMuscle in practice) for community-dwelling older adults: design and methods of a randomised controlled trial. BMC Public Health. (2018) 18:877. doi: 10.1186/s12889-018-5788-8, PMID: 30005654 PMC6045872

[32] van Dongen EJI Haveman-Nies A Doets EL Dorhout BG de Groot L. Effectiveness of a diet and resistance exercise intervention on muscle health in older adults: ProMuscle in practice. J Am Med Dir Assoc. (2020) 21:1065–72.e3. doi: 10.1016/j.jamda.2019.11.026, PMID: 31948853

[33] Dorhout BG de Groot LCPGM van Dongen EJI Doets EL Haveman-Nies A. Effects and contextual factors of a diet and resistance exercise intervention vary across settings: an overview of three successive ProMuscle interventions. BMC Geriatr. (2022) 22:189. doi: 10.1186/s12877-021-02733-6, PMID: 35264105 PMC8905865

[34] Reinders I Wijnhoven HAH Jyväkorpi SK Suominen MH Niskanen R Bosmans JE. Effectiveness and cost-effectiveness of personalised dietary advice aiming at increasing protein intake on physical functioning in community-dwelling older adults with lower habitual protein intake: rationale and design of the PROMISS randomised controlled trial. BMJ Open. (2020) 10:e040637. doi: 10.1136/bmjopen-2020-040637, PMID: 33444206 PMC7682452

[35] Reinders I Visser M Jyväkorpi SK Niskanen RT Bosmans JE Jornada Ben Â. The cost effectiveness of personalized dietary advice to increase protein intake in older adults with lower habitual protein intake: a randomized controlled trial. Eur J Nutr. (2022) 61:505–20. doi: 10.1007/s00394-021-02675-0, PMID: 34609621 PMC8490609

[36] Mertz KH Reitelseder S Bechshoeft R Bulow J Højfeldt G Jensen M. The effect of daily protein supplementation, with or without resistance training for 1 year, on muscle size, strength, and function in healthy older adults: a randomized controlled trial. Am J Clin Nutr. (2021) 113:790–800. doi: 10.1093/ajcn/nqaa372 33564844

[37] Weaver AA Tooze JA Cauley JA Bauer DC Tylavsky FA Kritchevsky SB. Effect of dietary protein intake on bone mineral density and fracture incidence in older adults in the health, aging, and body composition study. J Gerontol A Biol Sci Med Sci. (2021) 76:2213–22. doi: 10.1093/gerona/glab068, PMID: 33677533 PMC8599066

[38] Kemmler W Kohl M Jakob F Engelke K von Stengel S. Effects of high intensity dynamic resistance exercise and whey protein supplements on Osteosarcopenia in older men with low bone and muscle Mass. Final results of the randomized controlled FrOST study. Nutrients. (2020) 12:2341. doi: 10.3390/nu12082341 32764397 PMC7468852

[39] Health Council of the Netherlands. A healthy protein transition. The Hague: Health Council of the Netherlands (2023).

[40] Meltzer HM Brantsæter AL Trolle E Eneroth H Fogelholm M Ydersbond TA. Environmental sustainability perspectives of the Nordic diet. Nutrients. (2019) 11:2248. doi: 10.3390/nu11092248, PMID: 31540525 PMC6769495

[41] Schmidt P. Promoting healthy and sustainable diets in the EU European Economic and Social Comittee (2018). Brussels.

[42] Willett W Rockström J Loken B Springmann M Lang T Vermeulen S. Food in the Anthropocene: the EAT-lancet commission on healthy diets from sustainable food systems. Lancet. (2019) 393:447–92. doi: 10.1016/S0140-6736(18)31788-4, PMID: 30660336

[43] Tetens I Birt CA Brink E Bodenbach S Bugel S De Henauw S. Food-based dietary guidelines - development of a conceptual framework for future food-based dietary guidelines in Europe: report of a Federation of European Nutrition Societies Task-Force Workshop in Copenhagen, 12-13 march 2018. Br J Nutr. (2020) 124:1338–44. doi: 10.1017/s0007114520002469, PMID: 32624024

[44] Darling AL Millward DJ Lanham-New SA. Dietary protein and bone health: towards a synthesised view. Proc Nutr Soc. (2021) 80:165–72. doi: 10.1017/s0029665120007909, PMID: 33183359

[45] Domić J Grootswagers P van Loon LJC de Groot LCPGM. Perspective: vegan diets for older adults? A perspective on the potential impact on muscle mass and strength. Adv Nutr. (2022) 13:712–25. doi: 10.1093/advances/nmac009 35108354 PMC9156387

[46] Groenendijk I Grootswagers P Santoro A Franceschi C Bazzocchi A Meunier N. Protein intake and bone mineral density: cross-sectional relationship and longitudinal effects in older adults. J Cachexia Sarcopenia Muscle. (2023) 14:116–25. doi: 10.1002/jcsm.13111, PMID: 36346154 PMC9891984

[47] Iguacel I Miguel-Berges ML Gómez-Bruton A Moreno LA Julián C. Veganism, vegetarianism, bone mineral density, and fracture risk: a systematic review and meta-analysis. Nutr Rev. (2019) 77:1–18. doi: 10.1093/nutrit/nuy045, PMID: 30376075

[48] Tong TYN Appleby PN Armstrong MEG Fensom GK Knuppel A Papier K. Vegetarian and vegan diets and risks of total and site-specific fractures: results from the prospective EPIC-Oxford study. BMC Med. (2020) 18:353. doi: 10.1186/s12916-020-01815-3, PMID: 33222682 PMC7682057

[49] Gezondheidsraad. Gezonde eiwittransitie. (2023). Available at: https://www.gezondheidsraad.nl/onderwerpen/voeding/alle-adviezen-over-voeding/gezonde-eiwittransitie.

[50] van Dam CHJ Christensen SH Tetens I Riley III WF Timmer MJ Marin I. (2023). The co-creation of a digital tool that ensures sufficient protein quality in plant-based meals of older adults; a user-centered design approach. JMIR Publications. 10.2196/48323 PMC11695958 39700495

[51] Garlick PJ. The role of leucine in the regulation of protein metabolism. J Nutr. (2005) 135:1553s–6s. doi: 10.1093/jn/135.6.1553S 15930468

[52] Katsanos CS Kobayashi H Sheffield-Moore M Aarsland A Wolfe RR. A high proportion of leucine is required for optimal stimulation of the rate of muscle protein synthesis by essential amino acids in the elderly. Am J Physiol Endocrinol Metab. (2006) 291:E381–7. doi: 10.1152/ajpendo.00488.2005, PMID: 16507602

[53] Lv Z Shi W Zhang Q. Role of essential amino acids in age-induced bone loss. Int J Mol Sci. (2022) 23:11281. doi: 10.3390/ijms231911281, PMID: 36232583 PMC9569615

[54] Su Y Elshorbagy A Turner C Refsum H Chan R Kwok T. Circulating amino acids are associated with bone mineral density decline and ten-year major osteoporotic fracture risk in older community-dwelling adults. Bone. (2019) 129:115082. doi: 10.1016/j.bone.2019.115082, PMID: 31622772 PMC6925590

[55] Carbone L Bůžková P Fink HA Robbins JA Barzilay JI Elam RE. The Association of Tryptophan and its Metabolites with Incident hip Fractures, mortality, and prevalent frailty in older adults: the cardiovascular health study. JBMR Plus. (2023) 7:e10801. doi: 10.1002/jbm4.10801, PMID: 37808397 PMC10556266

[56] Grahnemo L Eriksson AL Nethander M Johansson R Lorentzon M Mellström D. Low circulating valine associate with high risk of hip fractures. J Clin Endocrinol Metab. (2023) 108:e1384–93. doi: 10.1210/clinem/dgad268, PMID: 37178220 PMC10583993

[57] Nishimura Y Højfeldt G Breen L Tetens I Holm L. Dietary protein requirements and recommendations for healthy older adults: a critical narrative review of the scientific evidence. Nutr Res Rev. (2023) 36:69–85. doi: 10.1017/S0954422421000329 34666855

[58] Traylor DA Gorissen SHM Phillips SM. Perspective: protein requirements and optimal intakes in aging: are we ready to recommend more than the recommended daily allowance? Adv Nutr. (2018) 9:171–82. doi: 10.1093/advances/nmy003, PMID: 29635313 PMC5952928

